# NEK8 promotes the progression of gastric cancer by reprogramming asparagine metabolism

**DOI:** 10.1186/s10020-024-01062-9

**Published:** 2025-01-06

**Authors:** Mingliang Wang, Kexun Yu, Futao Meng, Huizhen Wang, Yongxiang Li

**Affiliations:** General Surgery Department, The First Affiliated Hospital of Anhui Medical University, 218 Jixi Road, Hefei, China

**Keywords:** ASNS, Asparagine metabolism, NEK8, Ubiquitination

## Abstract

Several members of the NIMA-related kinase (NEK) family have been implicated in tumor progression; however, the role and underlying mechanisms of NEK8 in gastric cancer (GC) remain unclear. This study revealed a significant upregulation of NEK8 in GC, identifying it as an independent prognostic marker in patients with GC. Consistent with these findings, NEK8 silencing substantially impeded GC aggressiveness both in vitro and in vivo, while its overexpression produced the opposite effect. Gene Ontology enrichment analysis and metabolic profiling indicated that the impact of NEK8 on GC is primarily associated with reprogramming asparagine metabolism and modulating the mTORC1 pathway. Specifically, NEK8 knockdown suppressed asparagine synthesis by downregulating asparagine synthetase (ASNS) expression in GC cells. A strong correlation was observed between NEK8 levels and ASNS expression in human GC cells and tissue samples. Mechanistically, NEK8 directly interacts with ASNS, phosphorylating it at the S349 site, which inhibits its ubiquitination and subsequent degradation. Moreover, substituting the ASNS-S349 site with alanine abrogated the pro-tumorigenic effects of ASNS-WT overexpression. Additionally, asparagine was identified as an activator of the mTORC1 pathway, with reintroducing asparagine after NEK8 silencing restoring mTORC1 activity. Collectively, these findings demonstrate that NEK8-mediated asparagine synthesis and activation of the mTORC1 pathway play a critical role in promoting GC progression.

**Supplementary Information:**

The online version contains supplementary material available at 10.1186/s10020-024-01062-9.

## Introduction

Despite the advent of emerging perioperative treatments for gastric cancer (GC) (Smyth et al. 2020), it remains one of the most commonly diagnosed cancers and ranks as the fifth leading cause of cancer-related deaths worldwide (Bray et al. 2024). The median progression-free survival and overall survival for advanced GC remain under 12 months (Okunaka et al. 2023). Malignant cells, including GC cells, exhibit distinct metabolic characteristics compared to normal epithelial cells. The Warburg effect, initially proposing that cancer cells rely on aerobic glycolysis rather than oxidative phosphorylation (Warburg 1956), laid the foundation for the study of tumor metabolism. In addition to abnormal glucose metabolism, other altered metabolic processes, including the reprogramming of amino acid metabolism (Chen et al. 2024), support the unchecked growth and proliferation of GC cells. With the increasing understanding of metabolic reprogramming, cancer therapies targeting amino acid metabolism have recently been developed (Soon et al. 2024; Yamashita et al. 2021). Consequently, elucidating the mechanisms underlying amino acid metabolic alterations is essential for exploring novel therapeutic strategies and identifying potential targets in GC.

Asparagine (Asn), a non-essential amino acid synthesized from aspartate and glutamine through the action of asparagine synthetase (ASNS), has gained attention due to its role in cancer metabolism. Asn restriction has been an effective treatment for acute myeloid leukemia (ALL) due to the deficiency of ASNS in ALL cells (Gottschalk Højfeldt et al. 2021). However, solid tumors such as melanoma and pancreatic cancer (Taylor et al. 2001; Bachet et al. 2015), which also exhibit low ASNS expression, have shown resistance to Asn-limited therapy. Subsequent studies have sought to explain this resistance, revealing that transcription factors and kinases like activating transcription factor 4 (ATF4) and mechanistic target of rapamycin complex 1 (mTORC1) play pivotal roles in maintaining Asn homeostasis in cancer cells (Gwinn et al. 2018; Pathria et al. 2019). As a key enzyme in Asn synthesis, ASNS has been implicated in the progression of multiple malignancies (Chiu et al. 2019). Nevertheless, the role of ASNS and Asn metabolism in GC remains inadequately understood.

The NIMA-related kinase (NEK) family, characterized by a conserved serine/threonine kinase domain at the N-terminus, was initially recognized for its role in regulating mitosis (Panchal and Evan 2023). To date, eleven NEK family members have been identified in mammals, several of which play significant roles in cancer progression. For instance, NEK4 acts as an oncoprotein in lung cancer by modulating the EMT pathway (Ding et al. 2018a), while NEK2 enhances proliferation, migration, and invasion in GC by upregulating KDM5B expression (Li et al. 2019). The connection between NEK8 and tumor biology was first described in breast cancer (Bowers and Boylan 2004). However, the biological functions and mechanisms of NEK8 in most cancers, including GC, remain largely unexplored.

Our experiments demonstrated that NEK8 promotes GC progression primarily by reprogramming Asn metabolism. Mechanistically, NEK8 phosphorylates ASNS at the S349 site, preventing its ubiquitination and degradation. Elevated Asn levels subsequently activate the mTORC1 pathway. Previous studies by our team have shown that mTORC1 can regulate ASNS protein levels and, in turn, Asn levels (Wang et al. 2023). Therefore, the present study seeks to elucidate the functional mechanism of the ASNS/Asn/mTORC1/ASNS positive feedback loop and highlights NEK8 as a potential therapeutic target for Asn restriction therapy in GC.

## Materials and methods

### Cell culture, reagents, and antibodies

GES-1 normal gastric epithelial cells, along with the GC cell lines AGS, SGC7901, MGC803, and HGC27, were sourced from Genechem (Shanghai, China). The cells were cultured in RPMI-1640 medium (Corning, USA) supplemented with 10% FBS (Clark, USA), penicillin, and streptomycin (HyClone, USA). Protease and phosphatase inhibitors were procured from APExBIO, and PVDF membranes were obtained from Millipore. Additional reagents included Dynabeads™ Protein G, Lipofectamine 2000, Tween-20, PBS, and TBS, all from Invitrogen. The antibodies utilized in this study were as follows: the mTOR Substrates Antibody Sampler Kit from CST (Cat#9862 T), anti-NEK8 antibody from Sigma (Cat# HPA040679, RRID: AB_10796226), anti-Flag antibody from Sigma (Cat# F1804, RRID: AB_262044), ASNS antibody from Santa Cruz Biotechnology (Cat# sc-365809, RRID: AB_10843357), anti-p-ser antibody from Santa Cruz Biotechnology (Cat# sc-81514, RRID: AB_1128624), ubiquitin antibody from Proteintech (Cat# 10201-2-AP), and GAPDH antibody from Abcam (Cat# ab9485, RRID: AB_307275). Secondary antibodies were provided by CST (Cat#7074 T).

### Cell lentivirus infection

All lentiviruses used in this study, including those for NEK8 overexpression, NEK8 knockdown (RNAi), ASNS-WT, ASNS-S349A mutation, and ASNS-S349D mutation, were obtained from Genechem (Shanghai, China). For lentiviral infections, HGC27, MGC803, and SGC7901 cells were seeded in 6-well plates and allowed to adhere overnight (approximately 2 × 10^5^ cells/well). The following day, lentiviruses for NEK8, ASNS, and negative controls (NC) were added when the cells reached 30–50% confluency. Stable polyclonal GC cell lines expressing NEK8-OE, shNEK8, ASNS-WT, ASNS-S349A, and NC were established following 2 weeks of selection with puromycin (2 mg/ml). Overexpression and knockdown efficiency were assessed 72 h post-infection via immunoblotting. The shNEK8 sequences used were as follows: shNEK8#1: 5′-CGGGTGATTGCTACACTTT-3′, sh#2: 5′-TGGTGATC ATCAAGCAGAT-3′, sh#3: 5′-CCACCATTGTGGAGGCTTT-3′.

### Patients, tissue microarray, and immunohistochemistry

In this study, a tissue microarray (TMA) was constructed using 129 GC tissues and 24 randomly selected adjacent normal tissues, collected between 2006 and 2008 from the general surgery department of the First Affiliated Hospital of Anhui Medical University. All GC tissues were pathologically confirmed and staged according to the tumor-node-metastasis (TNM) staging system and the American Joint Committee on Cancer (7th edition). Immunohistochemical (IHC) staining was performed as previously described (Wang et al. 2021). The protein expression levels in the TMAs were independently evaluated by two pathologists, blinded to the patients' clinical information. This study received approval from the Ethics Committee of the First Affiliated Hospital of Anhui Medical University.

### RNA isolation and qPCR assays

Total RNA was extracted from GC tissues and cells using TRIzol (Invitrogen). Reverse transcription into cDNA was performed using the ReverTra Ace qPCR RT Master Mix (Toyobo), followed by real-time PCR with SYBR-Green mix (Toyobo) on an Applied Biosystems platform. Relative mRNA expression levels were calculated using the 2^−△△Ct^ method, with GAPDH serving as the endogenous control. All primers were synthesized by Invitrogen, with the specific sequences as follows: NEK8, F: 5′-GCTGCCAATGCTCAACACAG-3′ and R: 5′-CTTGATGGTCACA CCCGACT-3′; ASNS, F: 5′-GAGGAAGGCATTCAGGCTCT-3′ and R: 5′-CTGC GCGGAGAACATCAAAC-3′; GAPDH, F: 5′-ATCAAGAAGGTGGTGAAGCAG G-3′ and R: 5′-CGTCAAAGGTGGAGGAGTGG-3′.

### Western blot

Proteins from GC cell lines or tissues were extracted using the M-PER™ Mammalian Protein Extraction Reagent (Thermo, Cat#78501). Protein concentrations were quantified with the BCA Protein Assay Kit (Beyotime, P0012). Proteins were separated via SDS-PAGE and transferred to PVDF membranes. The membranes were blocked with TBST containing 5% milk for 1 h, followed by overnight incubation at 4 °C with primary antibodies. The next day, secondary antibodies were applied, and the membranes were visualized using enhanced chemiluminescence.

### Colony formation

Approximately 500 infected GC cells (HGC27, MGC803, or SGC7901) were seeded into 6-well plates. After 2 weeks of incubation, colonies were fixed with 4% paraformaldehyde for 20 min and stained with 1% crystal violet. The colonies were then counted and analyzed. Statistical significance was assessed using the Mann–Whitney U-test.

### Scratch-healing experiments

Cells (6 × 10^5^ per well) were cultured in 6-well plates and grown to confluence over 24 h. Once confluent, the medium was replaced with FBS-free RPMI-1640 after creating linear wounds. Wound closure was recorded at 0, 48, and 72 h using a Live Cell Station (Cell Discoverer 7, Zeiss).

### Transwell assays

For the Transwell invasion assays, Transwell chambers with 8 µm pore size (Costar) were placed into 24-well plates. The upper chambers were filled with diluted Matrigel matrix (BD Bioscience, USA) and 100 µL of infected GC cells suspended in a serum-free medium. To the lower chambers, 650 µL of RPMI-1640 medium containing 20% FBS, serving as a chemoattractant, was added. After 24 h, the chambers were fixed with 4% paraformaldehyde and stained with 1% crystal violet. Cells on the upper surface of the membrane were carefully removed, and the invaded cells were visualized and recorded using a Leica microscope. For the Transwell migration assays, chambers without Matrigel were used, with all other steps mirroring those of the invasion assay.

### Tumor xenograft experiments

For the xenograft model, 6-week-old male BALB/c nude mice were obtained from the Shanghai Experimental Animal Center and randomly divided into experimental and control groups (n = 6/group). Transfected GC cells (1 × 10^6^ cells in 100 µL per mouse) were injected subcutaneously into the lower area of the left upper limb. Tumor growth and general health were monitored weekly, with tumor size and volume measured every 4–5 days. When the tumor volume > 1.5 cm^3^, the mice were euthanized, and tumor growth curves were generated based on the collected data. Tumor tissues were then fixed in 4% paraformaldehyde for further analysis, including Hematoxylin & Eosin (H&E), IHC, and Tunel staining. All animal experiments and protocols were approved by the Anhui Medical University Ethics Committee.

### GC peritoneal cancer dissemination

To assess peritoneal cancer dissemination, 1 × 10^6^ infected GC cells were injected into the abdominal cavities of 4-week-old BALB/c nude mice. In vivo fluorescence imaging was employed to monitor peritoneal cancer dissemination over approximately 6 weeks. Firstly, the luciferase gene was stably integrated into the chromosomes of GC cells, and a cell line capable of stably expressing luciferase protein was cultured. The cell line was then injected intraperitoneally into mice. Before imaging, the mice were fasted for 4 h. After inhalation anesthesia, potassium fluorescein was injected intraperitoneally into the mice. During fluorescence imaging, luminescence was excited by injecting substrates, and the luminescence signal was detected using instruments for tumor imaging. Following this period, the mice were sacrificed, and the peritoneal cancer dissemination tumors were documented and analyzed.

### Co-immunoprecipitation

Co-immunoprecipitation was performed as previously described (Wang et al. 2023). Briefly, three transfected GC cell lines and corresponding control groups were prepared in advance and transferred to 100 mm culture dishes. After achieving 90–100% fusion, wash the cells with PBS and lyse them with pre-cooled M-PER protein lysis buffer at a concentration of 10^7^ cells/mL. Following centrifugation, collect the supernatant and divide it into three groups. One group was used as a positive control (input group), while the second group was added with homologous IgG as a blank control. The supernatant of the third group was mixed with NEK8 or ASNS monoclonal antibodies and Protein G beads, and an immunoprecipitation kit was used to form antigen antibody complexes. Cultivate the protein mixture on a rotating incubator at 4 °C for 4–5 h. After incubation, use a magnetic scaffold to precipitate G protein, primary antibody, and target protein complexes. Finally, protein complexes from all three groups were denatured at 95 °C and protein interactions were analyzed by Western blotting.

### LC–MS/MS

Liquid chromatography-tandem mass spectrometry (LC–MS/MS) was employed to analyze metabolic reprogramming in GC cells following NEK8 silencing. In brief, a precooled solution of methanol/acetonitrile/water (2:2:1, v/v) was added to the infected cells (approximately 2 × 10^6^ cells per sample). The mixture underwent low-temperature sonication for 30 min, followed by centrifugation at 14,000 g for 20 min. For LC–MS/MS analysis, the resulting supernatants were re-dissolved in 100 μL of acetonitrile/water (1:1, v/v). Separation was achieved using an Ultra High Pressure Liquid Chromatography (UHPLC, Agilent 1290 Infinity LC) HILIC column, and the primary and secondary spectra of the samples were acquired using a mass spectrometer (AB Triple TOF 6600). The raw MS data were analyzed to identify metabolite alterations in GC cells using XCMS software.

### Identification of ASNS phosphorylation sites and NEK8-binding proteins by MS

As previously described (Wang et al. 2023), HGC27 cells were transfected with a Flag-tagged NEK8 lentiviral plasmid. NEK8-binding proteins were pulled down using Flag M2 agarose beads (Sigma). The samples were washed with a buffer containing 20 mM Tris–HCl, 150 mM KCl, 1 mM dithiothreitol (DTT), 0.05% Nonidet P-40 (NP-40), 1 mM EDTA, 15% glycerol, and 0.2 mM PMSF. The pulled-down proteins were then separated via SDS-PAGE, excised, and subjected to MS analysis. Phosphorylation sites on ASNS were identified as previously described (Feng et al. 2020).

### Bioinformatic analysis

The expression of NEK8 in stomach cancer was analyzed using the UALCAN database (http://ualcan.path.uab.edu/index.html). RNA-sequencing (RNA-seq) datasets were downloaded from the TCGA website (https://portal.gdc.cancer.gov/), and data were processed using R version 3.6.1. The false discovery rate was calculated using the Benjamini–Hochberg procedure. Gene Ontology analysis was conducted using the tool (https://biit.cs.ut.ee/gprofiler/gost) to identify the top 500 upregulated genes. Additional NEK8 expression data in GC were sourced from the Oncomine database, a web-based cancer microarray database and data mining platform. GraphPad Prism 7.0 was used to visualize the NEK8 expression patterns.

### GST affinity isolation assay

As previously detailed(Feng et al. 2020), GST-tagged NEK8 or ASNS proteins were mixed with glutathione-Sepharose 4B beads (GE Healthcare, 17075601) and washed with an elution buffer containing 15 mM glutathione and 50 mM Tris–HCl (pH 8.0). The beads were then incubated with His-tagged ASNS or NEK8 proteins expressed in Escherichia coli BL21, followed by purification with Ni-nitrilotriacetic acid (NTA) agarose beads (Qiagen, 30,210) at 4 °C for five hours. The beads were washed with GST binding buffer (2 mM EDTA, 50 mM NaF, 100 mM NaCl, and 1% NP-40) (Thermo Fisher Scientific, 28324), and the bound proteins were eluted for subsequent immunoblotting.

### Protein stability assay

To assess protein stability after blocking or mimicking ASNS phosphorylation, a cycloheximide (CHX) chase assay was conducted. GC cells were transfected with ASNS-WT, ASNS-S349A, and ASNS-S349D plasmids, followed by treatment with 20 μg/mL CHX (MCE, Cat#HY-12320) for the indicated time points (0 h, 4 h, 8 h, and 12 h). Cell lysates were then collected and analyzed by Western blot.

### Statistical analysis

All experiments were repeated at least three times. Data were presented as mean ± standard deviation. All data were subjected to Student’s t-test or one-way analysis of variance (ANOVA) and otherwise processed with SPSS v. 22.0 (SPSS, Inc., Chicago, IL, USA) and GraphPad Prism 8.0 (GraphPad Software, La Jolla, CA, USA). The relationship between NEK8 expression and pathological variables was analyzed using Pearson's chi-squared test. Survival analysis was performed by the Kaplan‒Meier method with the log-rank test. For all tests, p < 0.05 was considered significant (ns: not significant, *p < 0.05, **p < 0.01, ***p < 0.001).

## Results

### NEK8 acts as an independent prognostic factor in patients with GC and promotes GC cell proliferation in vitro

**Fig. 1:**
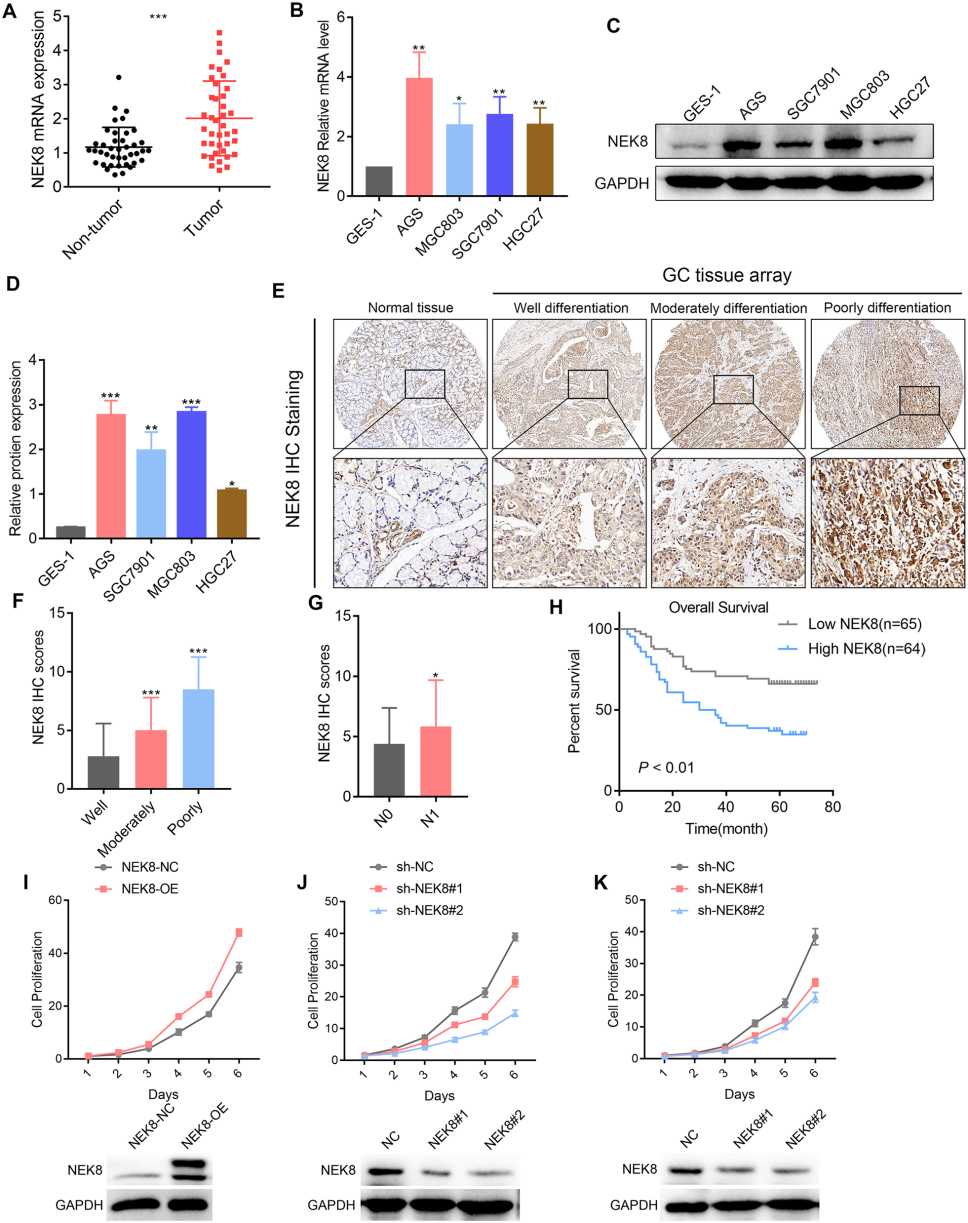
NEK8 acts as an independent prognostic factor and promotes GC cell proliferation in vitro. **A** NEK8 mRNA expression was significantly elevated in 40 pairs of GC tissues compared to adjacent normal tissues. **B**–**D** RT-PCR and Western blotting analyses revealed a significant upregulation of NEK8 mRNA and protein levels in GC cell lines. **E** Immunohistochemical analysis of a large-sample tissue microarray demonstrated that NEK8 was markedly overexpressed in GC tissues. **F**, **G** Tissue microarray analysis showed a significant correlation between NEK8 expression and both GC differentiation and lymph node metastasis. **H** Kaplan–Meier analysis indicated that NEK8 expression is significantly associated with poor prognosis in patients with GC. **I**, **J** NEK8 overexpression and knockdown experiments confirmed that NEK8 overexpression promotes GC cell proliferation, while NEK8 knockdown has the opposite effect. *P < 0.05, **P < 0.01, ***P < 0.001

To investigate NEK8 expression in GC, mRNA levels were first analyzed in forty pairs of GC tissues and adjacent normal tissues, revealing a significant upregulation of NEK8 in GC tissues compared to normal tissues (Fig. 1A). Subsequently, NEK8 protein and mRNA expression were evaluated across four GC cell lines (AGS, SGC7901, MGC803, and HGC27) as well as the GES-1 cell line, with results showing a marked increase in NEK8 levels in the GC cell lines relative to GES-1 (Fig. 1B–D). The clinical relevance of NEK8 was further explored using IHC staining on a tissue array comprising 153 samples (129 GC tissues and 24 normal tissues). The findings demonstrated a correlation between increased NEK8 expression and worsening GC differentiation, with IHC scores of 2.78, 5.02, and 8.50 for well, moderately, and poorly differentiated GC, respectively (Fig. 1E, F). Additionally, higher NEK8 expression was significantly associated with lymph node metastasis (Fig. 1G). Kaplan–Meier survival analysis revealed that patients with elevated NEK8 expression had poorer overall survival (OS) (Fig. 1H). Multivariate analysis confirmed NEK8 as an independent prognostic marker in GC (Table S1). To elucidate the biological function of NEK8 in GC, three short hairpin RNAs (shRNAs) targeting NEK8 and an overexpression lentivirus were developed. Based on NEK8 expression levels in GC cells, overexpression lentivirus was introduced into HGC27 cells, while shRNAs were transfected into SGC7901 and MGC803 cells. shNEK8#1 and shNEK8#2, which exhibited high inhibition rates, were selected for further experimentation. Cell counting assays indicated that NEK8 overexpression significantly enhanced GC cell proliferation, whereas NEK8 knockdown had the opposite effect (Fig. 1I, J). These data collectively suggest that NEK8 serves as an independent prognostic factor in patients with GC and promotes GC cell proliferation in vitro.

### NEK8 promotes GC cell clonal formation, wound healing, invasion, and migration in vitro

**Fig. 2:**
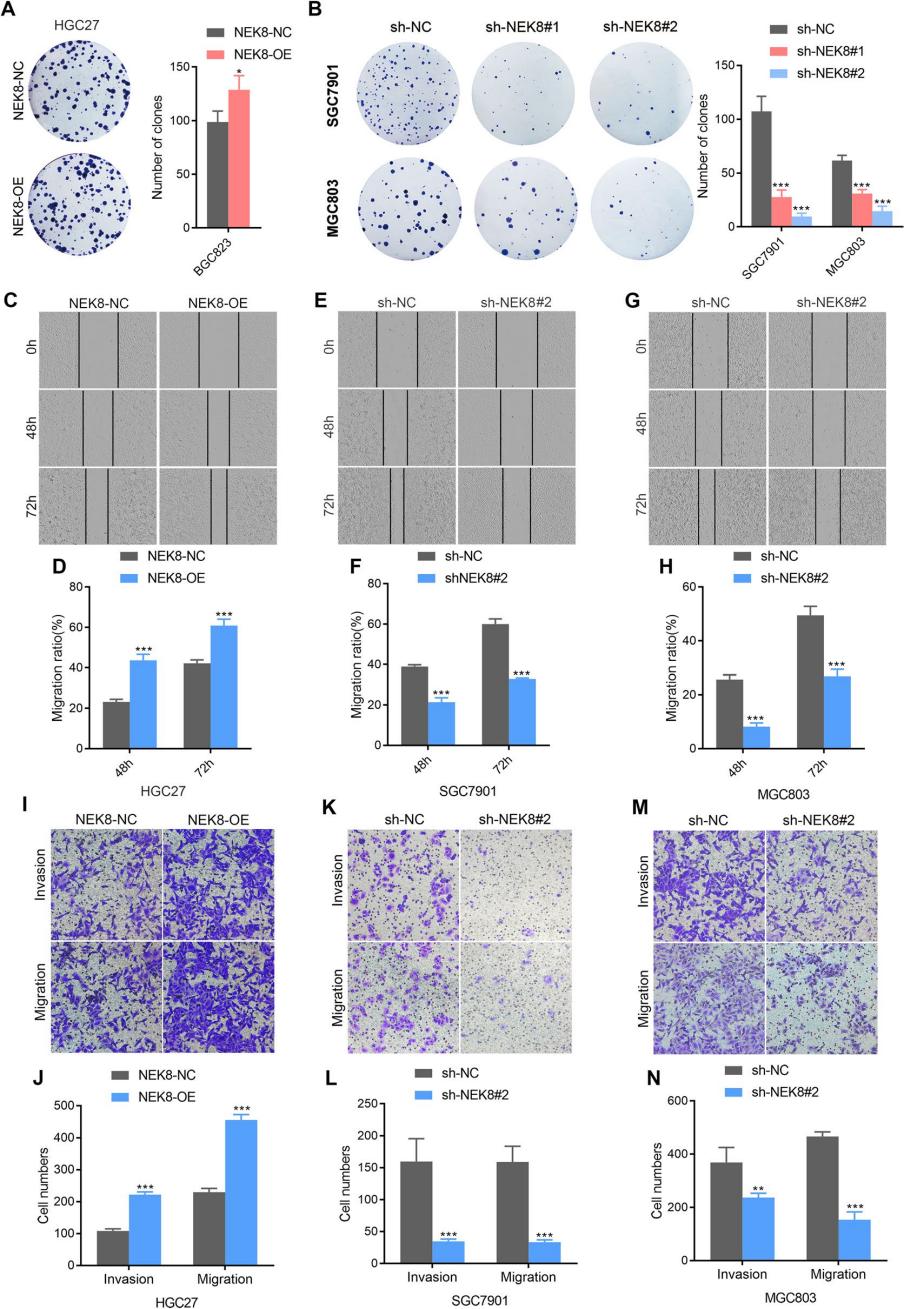
NEK8 promotes GC cell clonal formation, wound healing, invasion, and migration in vitro. **A** NEK8 overexpression enhanced the colony-forming capability of GC cells. **B** NEK8 knockdown significantly inhibited the colony-forming capability of GC cells. **C**, **D** Scratch healing assays showed that NEK8 overexpression markedly increased the horizontal migration capabilities of HGC27 cells. **E**–**H** Scratch healing assays further demonstrated that NEK8 knockdown significantly reduced the horizontal migration capabilities of SGC7901 and MGC803 cells. **I**, **J** NEK8 overexpression enhanced the invasive and migratory capabilities of HGC27 cells. **K**–**N** Transwell assays indicated that NEK8 knockdown significantly decreased the invasive and migratory capabilities of SGC7901 and MGC803 cells. *P < 0.05, **P < 0.01, ***P < 0.001

As described earlier, NEK8-overexpressing and NEK8-knockdown GC cells were further utilized to assess NEK8's impact on clonal formation, invasion, and migration capacity. The clonal formation assay demonstrated that NEK8 significantly enhanced the clonal formation ability of GC cells (Fig. 2A, B). Scratch-healing assays were performed to evaluate changes in horizontal migration, revealing that forced NEK8 expression increased horizontal migration, while NEK8 silencing produced the opposite effect (Fig. 2C–H). Transwell assays further confirmed that NEK8 overexpression in HGC27 cells enhanced both invasion and migration capabilities (Fig. 2I, J). Consistent with these results, NEK8 knockdown in SGC7901 and MGC803 cells markedly inhibited their invasion and migration abilities (Fig. 2K–N). Collectively, these results indicate that NEK8 promotes clonal formation, wound healing, invasion, and migration in GC.

### NEK8 facilitates GC progression and peritoneal cancer dissemination in vivo

**Fig. 3:**
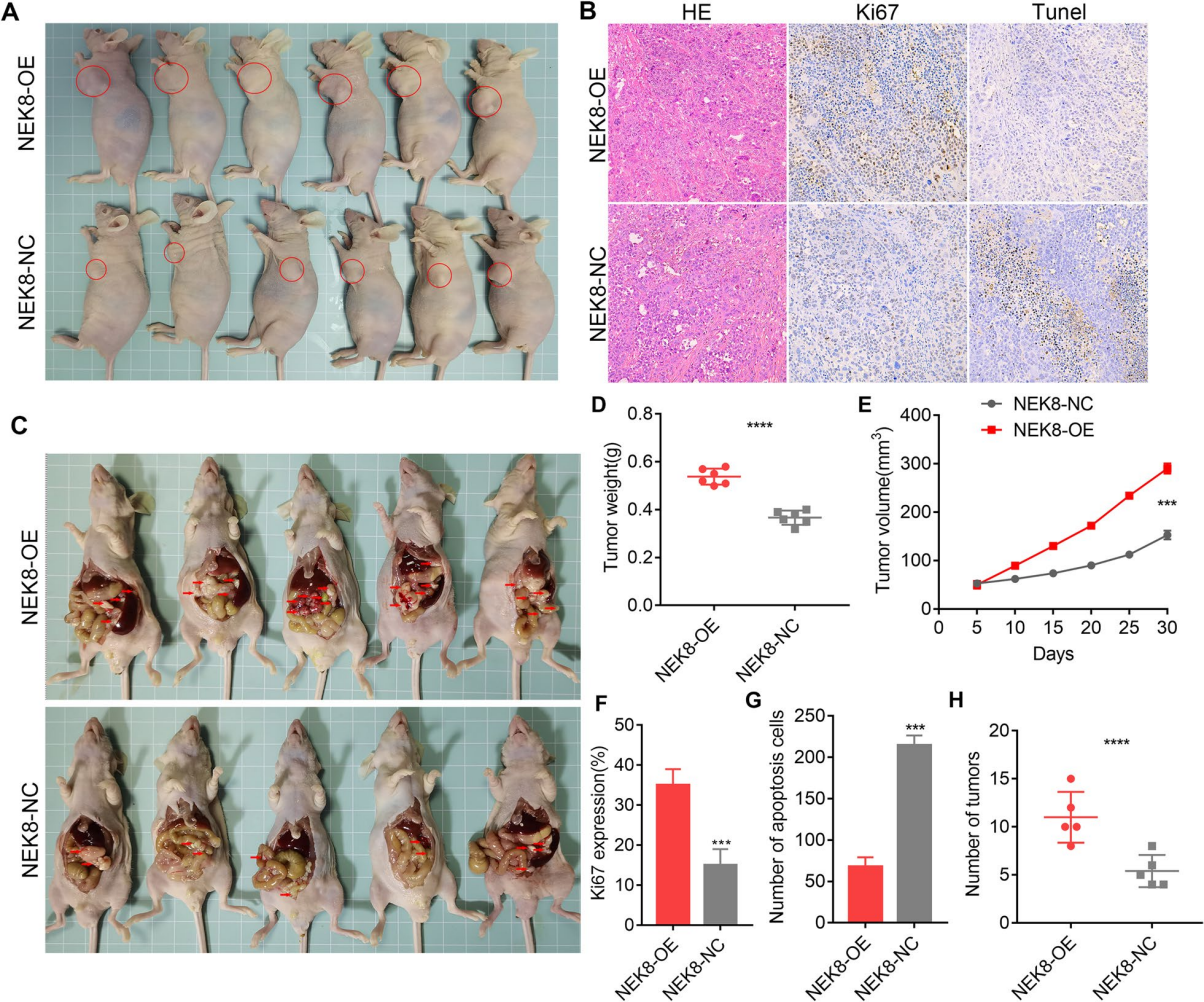
NEK8 facilitates GC progression and peritoneal cancer dissemination in vivo. **A** Tumor xenograft experiments demonstrated that NEK8 overexpression accelerates GC proliferation in vivo. **B** Ki-67 and TUNEL staining showed that NEK8 overexpression significantly increases the proliferation index while inhibiting GC apoptosis. **C** NEK8 overexpression promotes peritoneal cancer dissemination of GC cells. **D** Quantitative analysis of subcutaneous tumor weight. **E** Tumor growth curve of subcutaneous transplant. **F** Quantitative statistical analysis of the Ki-67 proliferation index. **G** Quantitative statistical analysis of the TUNEL staining index. **H** Quantitative analysis of the number of peritoneal cancer dissemination. *P < 0.05, **P < 0.01, ***P < 0.001

To further explore NEK8's role in GC progression and peritoneal cancer dissemination in vivo, subcutaneous tumor formation and peritoneal cancer dissemination assays were conducted. In Fig. 3A, we use circles to label the location of subcutaneous tumors in nude mice. At this point, the tumor xenograft experiments demonstrated that NEK8 overexpression accelerated GC proliferation and increased tumor volume in vivo (Fig. 3A, D, E). Ki-67 staining showed a significant increase in the proliferation index following NEK8 overexpression (Fig. 3B, F). TUNEL staining results indicated that NEK8 upregulation inhibited GC apoptosis (Fig. 3B, G). Additionally, infected cells were injected into the abdominal cavities of nude mice to assess whether NEK8 regulates GC peritoneal cancer dissemination in vivo. We specifically marked the peritoneal cancer dissemination nodules on the peritoneum and intestinal wall with arrows in Fig. 3C. The findings revealed that NEK8 overexpression promotes peritoneal cancer dissemination of GC cells (Fig. 3H). In summary, NEK8 facilitates GC progression and peritoneal cancer dissemination in vivo.

### NEK8 regulates amino acid metabolism and the mTORC1 pathway

**Fig. 4:**
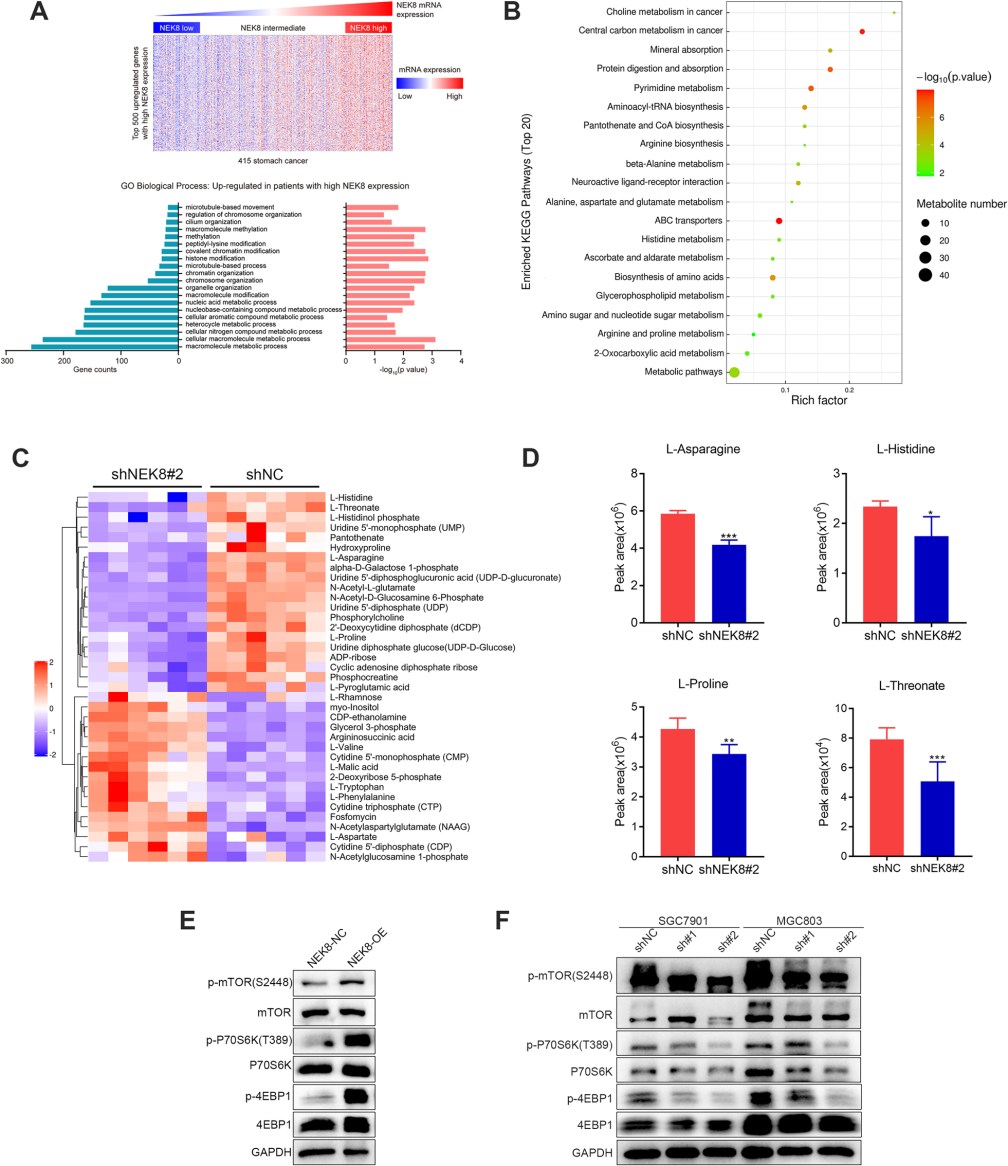
NEK8 regulates amino acid metabolism and the mTORC1 pathway. **A** Gene Ontology enrichment analysis revealed that high NEK8 expression primarily impacts tumor metabolism pathways. **B**, **C** Bubble chart and hierarchical clustering analysis show that NEK8 silencing significantly inhibits the metabolism of multiple amino acids. **D** NEK8 silencing notably reduces the levels of L-asparagine, L-histidine, L-proline, and L-threonate. **E**, **F** NEK8 overexpression upregulates the phosphorylation levels of mTOR (S2448), P70S6K (T389), and 4EBP1, while NEK8 knockdown exerts the opposite effect. *P < 0.05, **P < 0.01, ***P < 0.001

To elucidate the mechanisms by which NEK8 influences GC progression, Gene Ontology enrichment analysis was conducted to identify the top 20 biological processes associated with elevated NEK8 expression. The results indicated that high NEK8 levels predominantly impact tumor metabolism pathways (Fig. 4A). Further investigation using liquid chromatography-tandem mass spectrometry (LC–MS/MS) revealed that NEK8 silencing significantly downregulated the metabolic concentrations of several amino acids, including L-asparagine, L-histidine, L-proline, and L-threonate (Fig. 4B–D).

Given mTORC1's critical role in maintaining amino acid homeostasis, phosphorylation levels of mTOR and its downstream targets were assessed following NEK8 overexpression and knockdown. NEK8 was found to upregulate p-mTOR (S2448), p-P70S6K (T389), and p-4EBP1 (Fig. 4E, F), suggesting that NEK8 may modulate the mTORC1 pathway by enhancing amino acid metabolism. These results collectively indicate that NEK8 regulates both amino acid metabolism and the mTORC1 pathway.

### ASNS serves as a downstream molecule of NEK8

**Fig. 5:**
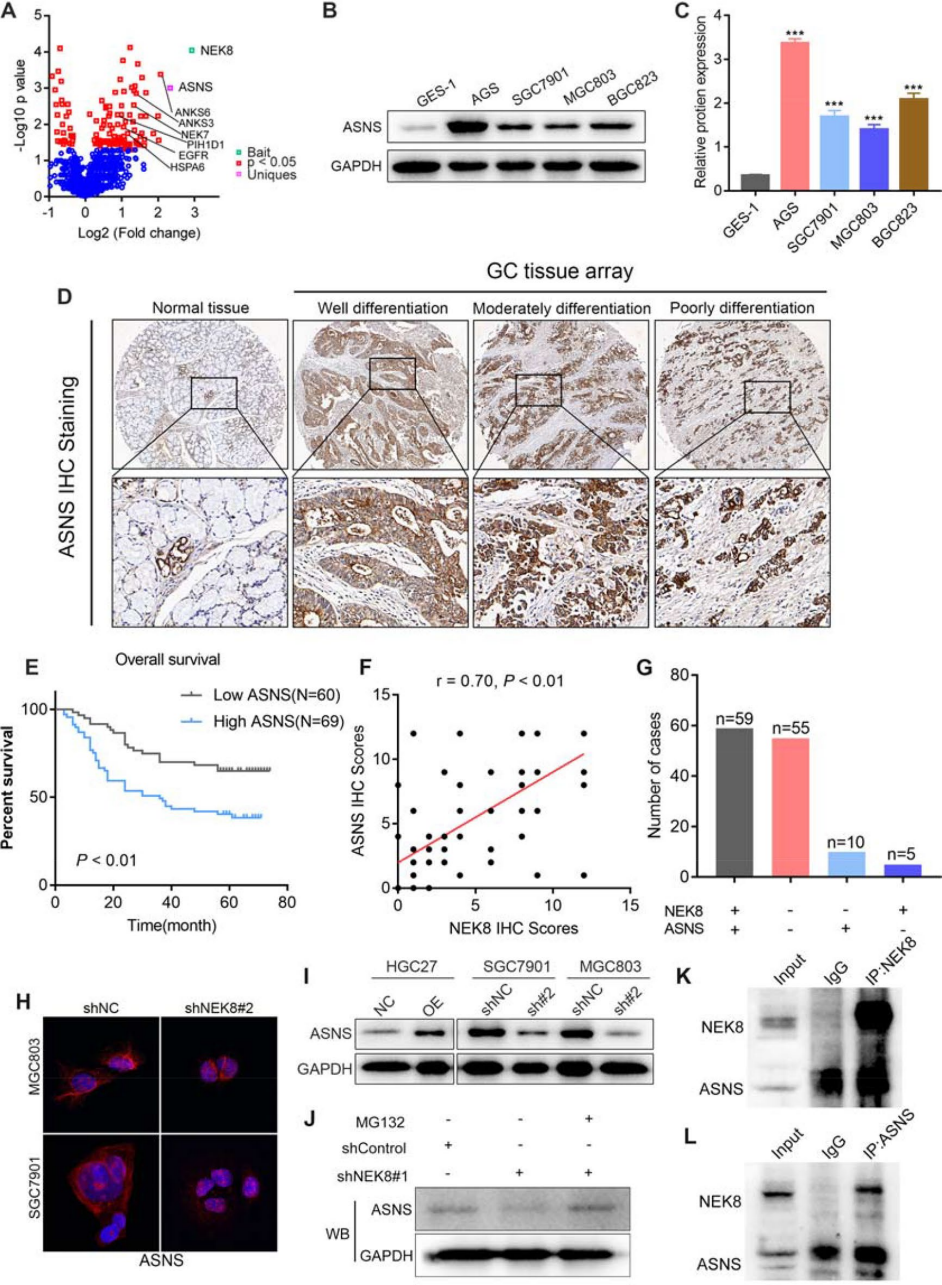
ASNS serves as a downstream molecule of NEK8. **A** IP-MS analysis revealed a significant correlation between NEK8 and ASNS. **B**, **C** ASNS levels were significantly elevated in GC cells compared to GES-1 cells. **D** Immunohistochemical analysis of a large-sample tissue microarray showed that ASNS was markedly overexpressed in GC tissues. **E** Kaplan–Meier survival analysis indicated that high ASNS expression is associated with a worse prognosis in patients with GC. **F**, **G** Pearson correlation analysis demonstrated a positive relationship between NEK8 expression and ASNS IHC staining. **H**, **I** IF and Western blot analyses confirmed that NEK8 significantly regulates ASNS protein expression. **J** The inhibition of ASNS protein expression by NEK8 knockdown was reversed by treatment with MG132. **K** NEK8 protein was pulled down using specific antibody and then ASNS protein was detected in the immunoprecipitated protein lysate of NEK8. **L** Subsequently, ASNS was also pulled down, and NEK8 could also be detected in the immunoprecipitated protein lysate of ASNS. *P < 0.05, **P < 0.01, ***P < 0.001

To further investigate how NEK8 inhibits amino acid metabolism in GC cells, mass spectrometry was performed following NEK8 immunoprecipitation (IP-MS). This analysis identified several proteins significantly correlated with NEK8, including ASNS, a key enzyme in asparagine synthesis (Fig. 5A). Based on these results, it was hypothesized that NEK8 might regulate L-asparagine metabolism via ASNS.

Subsequent analysis showed that ASNS expression was significantly elevated in GC cells compared to GES-1 cells (Fig. 5B, C). Similar findings were observed in GC tissue microarray analysis (Fig. 5D). Kaplan–Meier survival analysis indicated that high ASNS expression in patients with GC was associated with a poorer prognosis (Fig. 5E). Pearson correlation analysis demonstrated a positive relationship between NEK8 expression and ASNS IHC staining (Fig. 5F, G). Immunofluorescence and Western blot analyses confirmed that NEK8 significantly upregulates ASNS protein expression (Fig. 5H, I). Treatment of NEK8 knockdown cell lines with MG132 revealed that inhibiting ASNS ubiquitination mitigated the reduction in ASNS protein expression caused by NEK8 knockdown (Fig. 5J). Co-immunoprecipitation in AGS cells further validated the interaction between NEK8 and ASNS (Fig. 5K, L). These results suggest that ASNS may function as a downstream effector of NEK8, with NEK8 upregulating ASNS expression by inhibiting its ubiquitination.

### NEK8 directly interacts with and probably phosphorylates ASNS at the S349 site

**Fig. 6:**
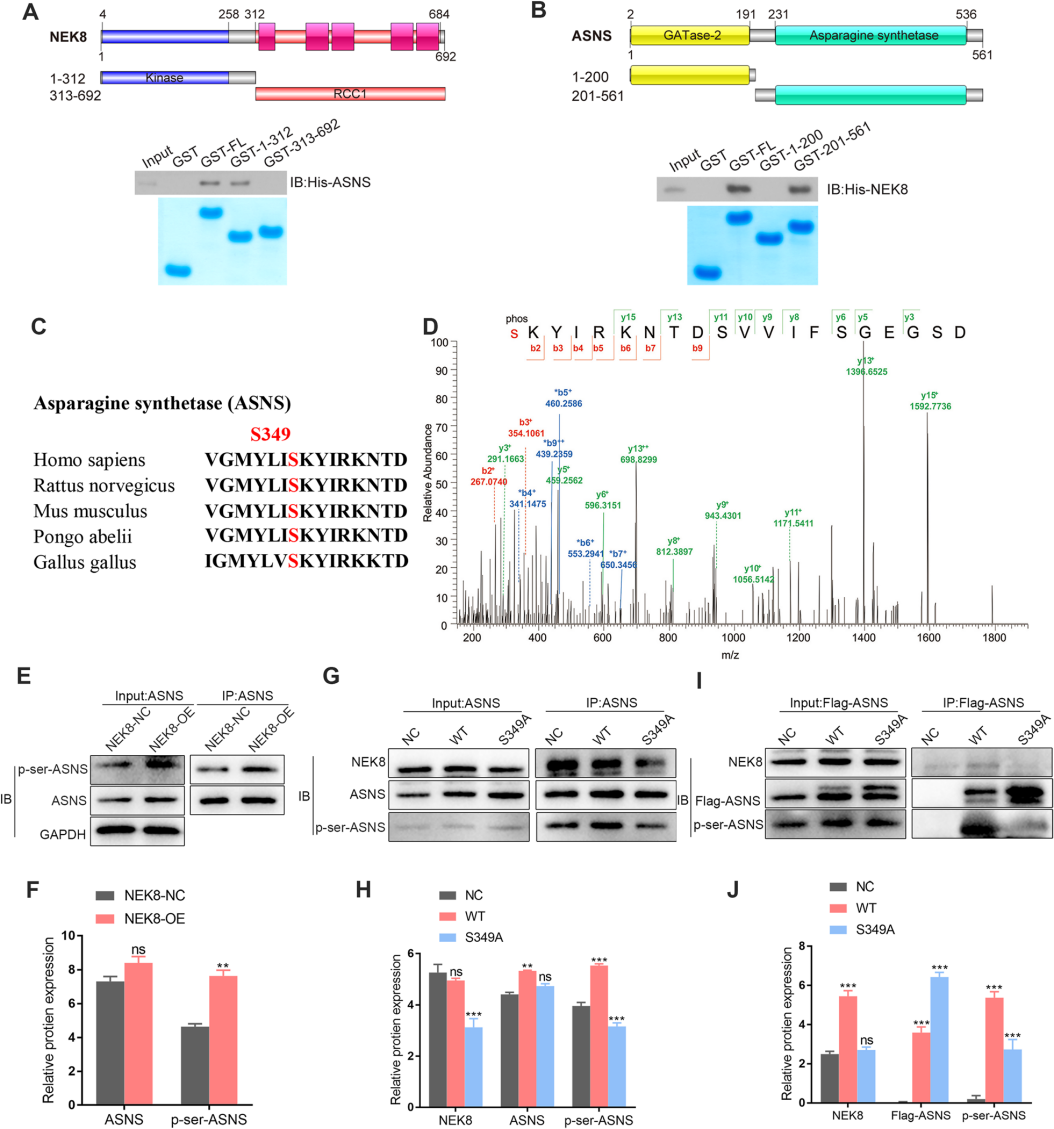
NEK8 directly interacts with and phosphorylates ASNS at the S349 site. **A** GST affinity isolation assays demonstrated that the NEK8 kinase domain, but not the RCC1 domain, interacts with ASNS. **B** His-NEK8 was shown to interact with the FL and the asparagine synthetase domain of GST-ASNS, but not with GST alone or the GATase-2 domain of ASNS. **C** Depiction of the highly conserved motif of ASNS. **D** Mass spectrometry (MS) identified the ASNS peptide containing the S349 site via NEK8 immunoprecipitation. **E**, **F** Phosphorylation of ASNS was detected using an anti-p-serine antibody following NEK8 overexpression, indicating that NEK8 promotes serine phosphorylation of ASNS. **G**–**J** Co-immunoprecipitation assays revealed that WT-ASNS/Flag-ASNS, but not ASNS-S349A/Flag-ASNS-S349A, was able to interact with NEK8. *P < 0.05, **P < 0.01, ***P < 0.001

As previously noted, NEK8 and ASNS interact with each other. To further identify the specific domain of ASNS involved in NEK8 binding, a GST affinity isolation assay was conducted. Constructs were created for full-length (FL) and two deletion fragments of GST-ASNS and GST-NEK8, respectively, followed by GST affinity isolation assays. The results demonstrated that the NEK8 kinase domain, but not the RCC1 domain, interacted with ASNS (Fig. 6A). Additionally, it was shown that His-NEK8 binds to the FL and the asparagine synthetase domain of GST-ASNS, but not to GST alone or the GATase-2 domain of ASNS (Fig. 6B). These results confirm that NEK8 directly interacts with ASNS.

As a member of the NEK family, NEK8 contains a conserved serine/threonine kinase domain in the N-terminal region (Panchal and Evan 2023). Given the interaction between NEK8 and ASNS, it was hypothesized that NEK8 directly phosphorylates ASNS and that this phosphorylation modulates ASNS function. To investigate this, the highly conserved ASNS motif across various metazoans was analyzed (Fig. 6C). Mass spectrometry identified that the ASNS peptide containing the S349 site was recognized by NEK8 during immunoprecipitation (Fig. 6D). A significant increase in ASNS phosphorylation was detected using an anti-p-serine antibody following NEK8 overexpression (Fig. 6E, H). Co-immunoprecipitation assays further revealed that WT-ASNS/Flag-ASNS, but not the ASNS-S349A/Flag-ASNS-S349A mutant, could interact with NEK8 (Fig. 6F, G, I, J). Collectively, these results indicate that NEK8 directly binds to ASNS and probably phosphorylates ASNS at the S349 site, thereby regulating the biological progression of GC.

### Phosphorylation of ASNS inhibits ubiquitination degradation and promotes the proliferation, invasion, and migration of GC cells

**Fig. 7:**
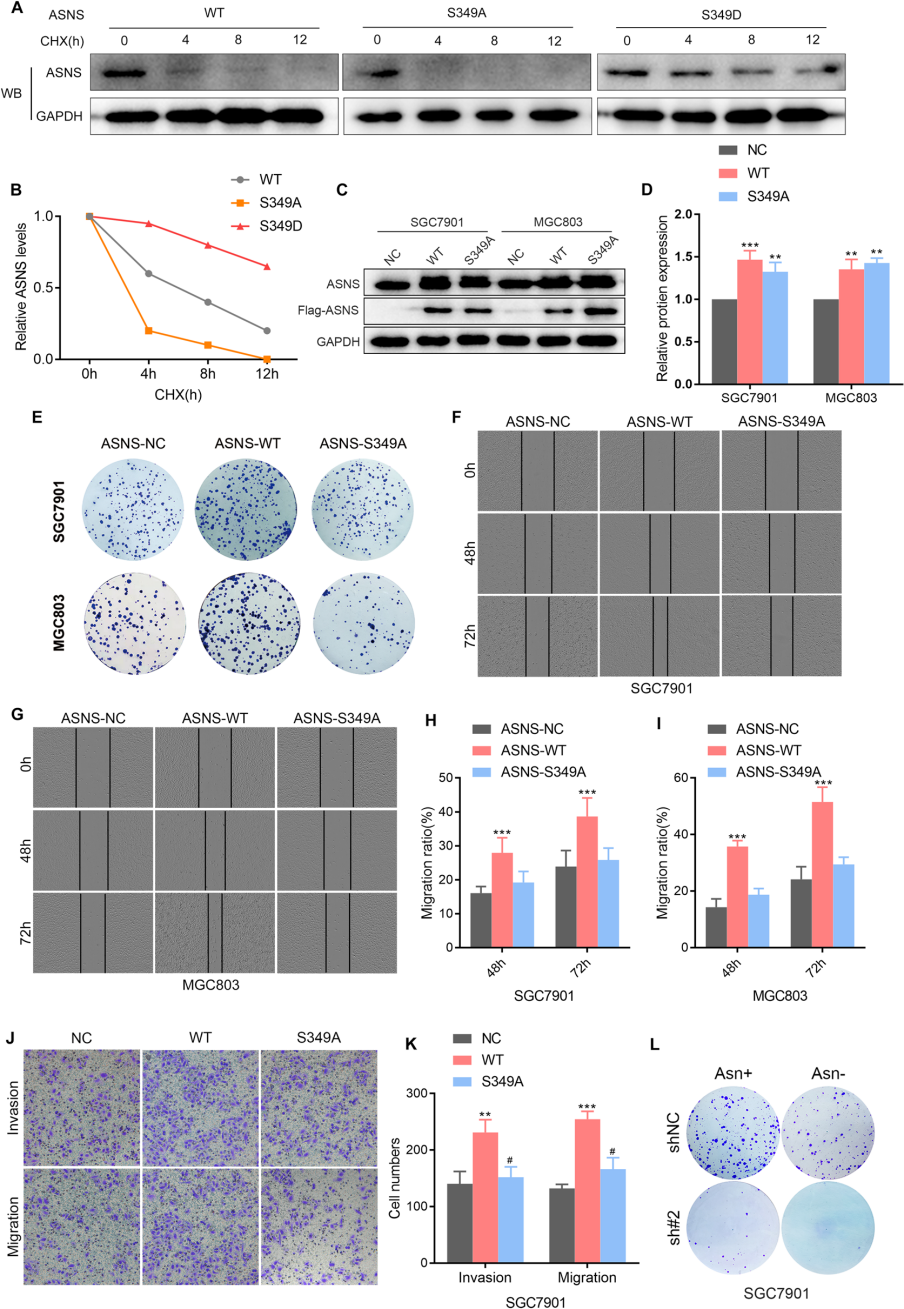
Phosphorylation of ASNS inhibits ubiquitination degradation and promotes proliferation, invasion, and migration of GC cells. **A**, **B** ASNS-S349A promotes the ubiquitination and degradation of ASNS, whereas ASNS-S349D has the opposite effect. **C**, **D** Western blot confirmed the overexpression efficiency of ASNS-WT and ASNS-S349A. **E** Overexpression of ASNS-WT enhanced the colony-forming capability of GC cells, while ASNS-S349A did not. **F**–**I** WT-ASNS, but not ASNS-S349A, significantly promoted wound healing capacity. **J**, **K** WT-ASNS, but not ASNS-S349A, promoted invasion and migration in GC cells. **L** Inhibition of colony formation was more pronounced under Asn-deprived conditions when combined with NEK8 silencing compared to NEK8 silencing alone. *P < 0.05, **P < 0.01, ***P < 0.001

To investigate the functional role of the ASNS S349 site, a site-specific mutation was employed, substituting serine 349 with alanine to prevent NEK8-mediated phosphorylation and with aspartic acid to mimic phosphorylation. GC cells were transfected with ASNS-WT, ASNS-S349A, and ASNS-S349D plasmids after reducing the endogenous expression of ASNS, and ASNS protein degradation was assessed following MG132 treatment at 0 h, 4 h, 8 h, and 12 h intervals. The results demonstrated that ASNS-S349A enhances the ubiquitination and degradation of ASNS, whereas ASNS-S349D exhibits the opposite effect (Fig. 7A, B). The overexpression efficiency of ASNS-WT and ASNS-S349A was confirmed via Western blot (Fig. 7C, D), followed by in vitro functional assays. These experiments revealed that while forced expression of WT-ASNS significantly promoted colony formation, wound healing, migration, and invasion, ASNS-S349A did not (Fig. 7E–K). Further clonal formation experiments were conducted under varying concentrations of asparagine (50 nM vs. 500 nM) following NEK8 silencing. The results indicated that the inhibition of colony formation was more pronounced under asparagine-deprived conditions when combined with NEK8 silencing, compared to NEK8 silencing alone (Fig. 7L). Additionally, it was confirmed that asparagine can independently activate the mTORC1 pathway (Figure S1) and that NEK8 modulates the mTORC1 pathway by regulating asparagine metabolism (Figure S2). Collectively, these results suggest that phosphorylation of ASNS at S349 inhibits its ubiquitination and degradation, thereby promoting GC cell proliferation, invasion, and migration. Moreover, the upregulation of asparagine activates the mTORC1 pathway, leading to the conclusion that the ASNS/Asn/ mTORC1/ASNS axis functions as a positive feedback loop, driving GC progression.

## Discussion

**Fig. 8:**
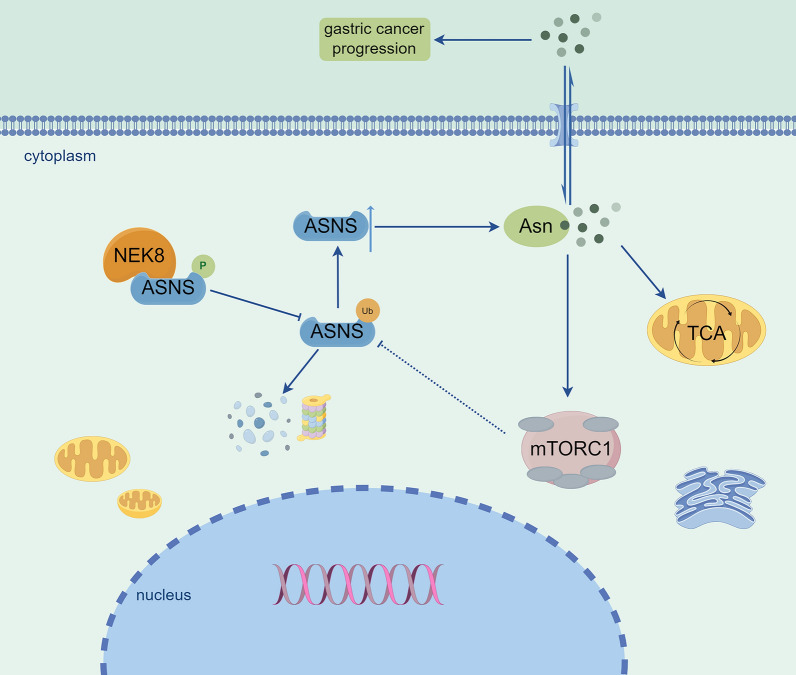
Illustration of the mechanism by which NEK8 kinase enhances GC progression through the reprogramming of Asn metabolism

The present study elucidated the role of NEK8 kinase in reprogramming asparagine (Asn) metabolism in GC (Fig. 8). Our findings demonstrated the following: (i) NEK8 is overexpressed in GC and strongly correlates with patient prognosis, while NEK8 silencing significantly inhibits GC cell aggressiveness both in vitro and in vivo; (ii) the effect of NEK8 in GC is significantly associated with Asn metabolism reprogramming and the mTORC1 pathway; (iii) NEK8 directly phosphorylates ASNS at the S349 site, thereby upregulating ASNS protein levels; (iv) phosphorylation at the ASNS S349 site inhibits ubiquitination and degradation, promoting GC cell proliferation, invasion, and migration; and (v) Asn can independently activate the mTORC1 pathway. Collectively, our results clarify the functional significance of the NEK8/ASNS/Asn/mTORC1/ASNS positive feedback loop in GC progression, a mechanism that had not been previously explored. Therefore, our study provides a new theoretical basis for targeted therapy in GC.

Previous studies have identified several tumor-associated kinases as crucial in GC, including CDKs (Zhao et al. 2020), the SRC kinase family (Poh et al. 2020), and the PI3K/AKT/mTOR kinase pathway (Fattahi et al. 2020). However, the role of the NEK family in GC remains largely unexplored. A recent pilot study on stomach cancer suggested that NEK8 promotes GC cell proliferation (Ding et al. 2018b), but the downstream targets and mechanisms of NEK8 in carcinogenesis were not addressed. In this report, we demonstrated that NEK8 markedly enhances GC cell growth and dissemination both in vitro and in vivo. Furthermore, multivariate analyses revealed that NEK8 expression is an independent prognostic indicator in GC. These data collectively suggest that NEK8 functions as an oncogene in GC and could serve as a potential therapeutic target.

Reprogramming of energy metabolism is increasingly recognized as a hallmark of cancer (Hanahan et al . 2011). In recent years, amino acid metabolism has garnered growing attention in cancer research and treatment (Yang et al. 2023; Li and Zhang 2024; Jin et al. 2023). While glutamine metabolism in stomach cancer has been extensively studied (Li and Zhang 2016; Ye et al. 2018), the role of Asn metabolism in GC remains poorly understood. This study focused on the post-translational modification of ASNS, a key enzyme in Asn synthesis, including its ubiquitination and phosphorylation. Our data identified ASNS as a novel substrate of NEK8. We found that NEK8-mediated phosphorylation of ASNS inhibits its ubiquitination and degradation, leading to the upregulation of intracellular Asn levels and subsequent activation of the mTORC1 pathway. Therefore, our findings uncover the critical role of the NEK8-ASNS-Asn-mTORC1 axis in GC, highlighting a previously unrecognized molecular mechanism that could pave the way for novel therapeutic strategies in GC.

Our observations indicated that ASNS-S349A, but not ASNS-S349D or ASNS-WT, is susceptible to ubiquitin-mediated recognition and degradation, underscoring the critical role of ASNS-S349 phosphorylation in maintaining intracellular Asn levels. Targeting Asn metabolism is a well-established strategy for treating acute lymphoblastic lymphoma (Gottschalk Højfeldt et al. 2021; Chan et al. 2019); however, Asn limitation has not produced the expected therapeutic outcomes in melanoma and pancreatic cancer due to compensatory mechanisms via the MAPK pathway (Pathria et al. 2019). Thus, exploring methods to inhibit ASNS levels could open new avenues for Asn-restricted therapies. In this study, we proposed that blocking ASNS phosphorylation could enhance its ubiquitination and degradation, suggesting that the S349 residue of ASNS may serve as an effective therapeutic target for patients with GC exhibiting elevated NEK8 expression. This conclusion is supported by the following data: (1) NEK8 overexpression increases pS349-ASNS phosphorylation; (2) the phosphorylation status of the S349 residue influences ASNS ubiquitination and degradation, thereby dictating Asn metabolism; and (3) combining Asn restriction with NEK8 knockdown more effectively inhibits GC cell proliferation than Asn restriction alone.

This study offers a strategic approach to investigating tumor metabolism. Both our current and previous findings reveal a close association between tumor-associated kinases and amino acid metabolism in tumor cells (Wang et al. 2023). For example, CDC-like kinase 3 (CLK3) plays a similar role in cholangiocarcinoma cells (Zhou et al. 2020), and well-known oncogenic kinases such as phosphoinositide 3-kinase (PI3K)/AKT are linked to metabolic gene expression and enzyme activity (Park et al. 2020). However, such studies remain limited in GC, highlighting the need for further research into the potential connections between tumor-associated kinases and cell metabolism in this cancer type.

In summary, this study provides compelling evidence that NEK8 contributes to Asn metabolism reprogramming by regulating ASNS, Asn, and the mTORC1 pathway in GC. These insights strongly support the potential of NEK8 as a novel therapeutic target in GC and suggest a new approach to treating this aggressive and invasive disease.

## Supplementary Information

Supplementary material 1: Figure S1 Asn independently activates the mTORC1 pathway.

Supplementary material 2: Figure S2 NEK8 regulates the mTORC1 pathway by modulating Asn metabolism.

Supplementary material 3.

## Data Availability

No datasets were generated or analysed during the current study.
