# Correction: NEK8 promotes the progression of gastric cancer by reprogramming asparagine metabolism

**DOI:** 10.1186/s10020-025-01234-1

**Published:** 2025-05-10

**Authors:** Mingliang Wang, Kexun Yu, Futao Meng, Huizhen Wang, Yongxiang Li

**Affiliations:** General Surgery Department, The First Affiliated Hospital of Anhui Medical University, 218 Jixi Road, Hefei, China

**Correction: Mol Med 31, 3 (2025)**

**https://doi.org/10.1186/s10020-024-01062-9**

In this article (Wang et al. 2025) the wrong figure appeared in Fig. 5K and have now been corrected in the original publication. For completeness and transparency, both correct and incorrect versions are displayed below.

The original article has been corrected.

Incorrect Fig. 5

**Fig. 5:**
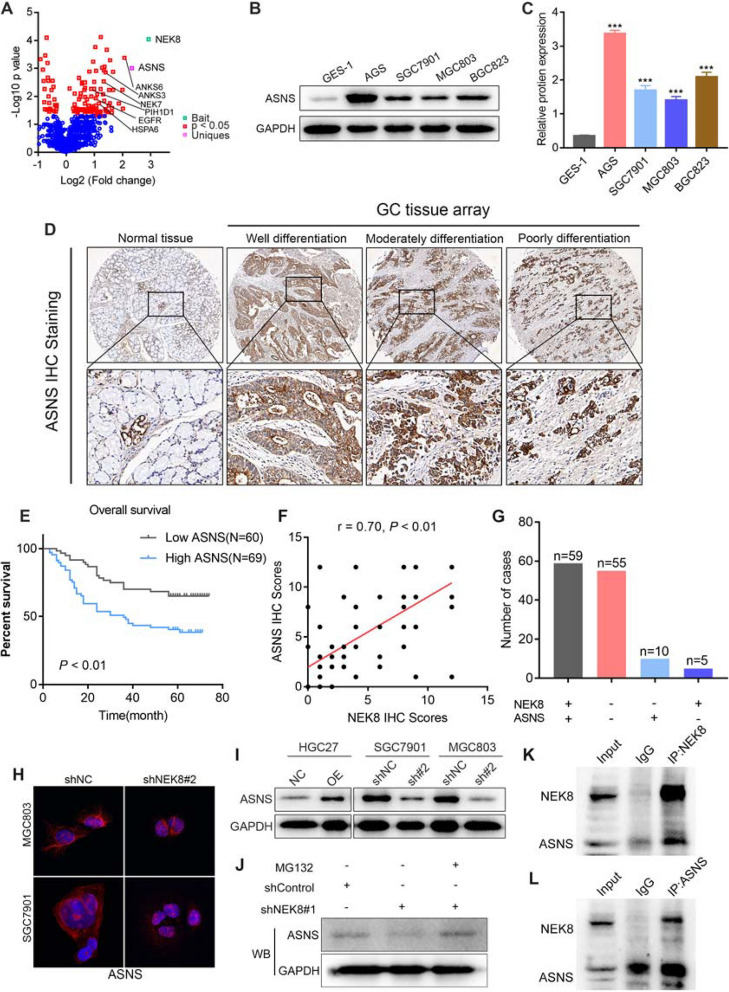
ASNS serves as a downstream molecule of NEK8. **A** IP-MS analysis revealed a significant correlation between NEK8 and ASNS. **B**, **C** ASNS levels were significantly elevated in GC cells compared to GES-1 cells. **D** Immunohistochemical analysis of a large-sample tissue microarray showed that ASNS was markedly overexpressed in GC tissues. **E** Kaplan–Meier survival analysis indicated that high ASNS expression is associated with a worse prognosis in patients with GC. **F**, **G** Pearson correlation analysis demonstrated a positive relationship between NEK8 expression and ASNS IHC staining. **H**, **I** IF and Western blot analyses confirmed that NEK8 significantly regulates ASNS protein expression. **J** The inhibition of ASNS protein expression by NEK8 knockdown was reversed by treatment with MG132. **K** NEK8 protein was pulled down using specific antibody and then ASNS protein was detected in the immunoprecipitated protein lysate of NEK8. **L** Subsequently, ASNS was also pulled down, and NEK8 could also be detected in the immunoprecipitated protein lysate of ASNS. *P < 0.05, **P < 0.01, ***P < 0.001

Correct Fig. 5

**Fig. 5:**
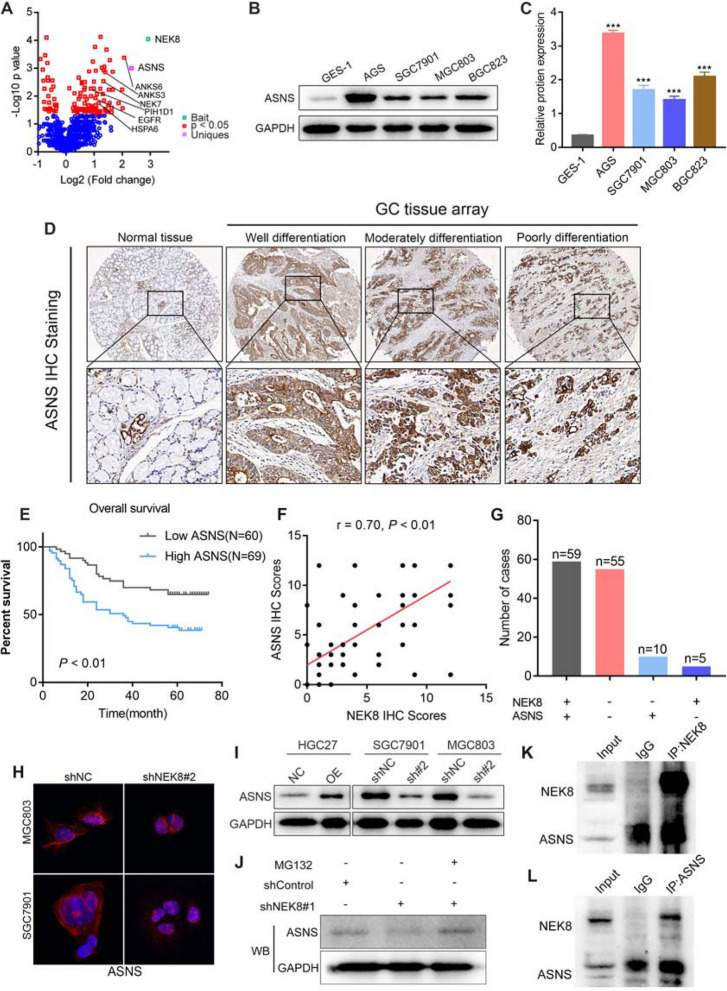
ASNS serves as a downstream molecule of NEK8. **A** IP-MS analysis revealed a significant correlation between NEK8 and ASNS. **B**, **C** ASNS levels were significantly elevated in GC cells compared to GES-1 cells. **D** Immunohistochemical analysis of a large-sample tissue microarray showed that ASNS was markedly overexpressed in GC tissues. **E** Kaplan–Meier survival analysis indicated that high ASNS expression is associated with a worse prognosis in patients with GC. **F**, **G** Pearson correlation analysis demonstrated a positive relationship between NEK8 expression and ASNS IHC staining. **H**, **I** IF and Western blot analyses confirmed that NEK8 significantly regulates ASNS protein expression. **J** The inhibition of ASNS protein expression by NEK8 knockdown was reversed by treatment with MG132. **K** NEK8 protein was pulled down using specific antibody and then ASNS protein was detected in the immunoprecipitated protein lysate of NEK8. **L** Subsequently, ASNS was also pulled down, and NEK8 could also be detected in the immunoprecipitated protein lysate of ASNS. *P < 0.05, **P < 0.01, ***P < 0.001
