# Supplementary figures and images for: NEK8 promotes the progression of gastric cancer by reprogramming asparagine metabolism
Source: Mol Med. 2025 Jan 6;31:3. doi: 10.1186/s10020-024-01062-9 (PMC11702068; doi:10.1186/s10020-024-01062-9)

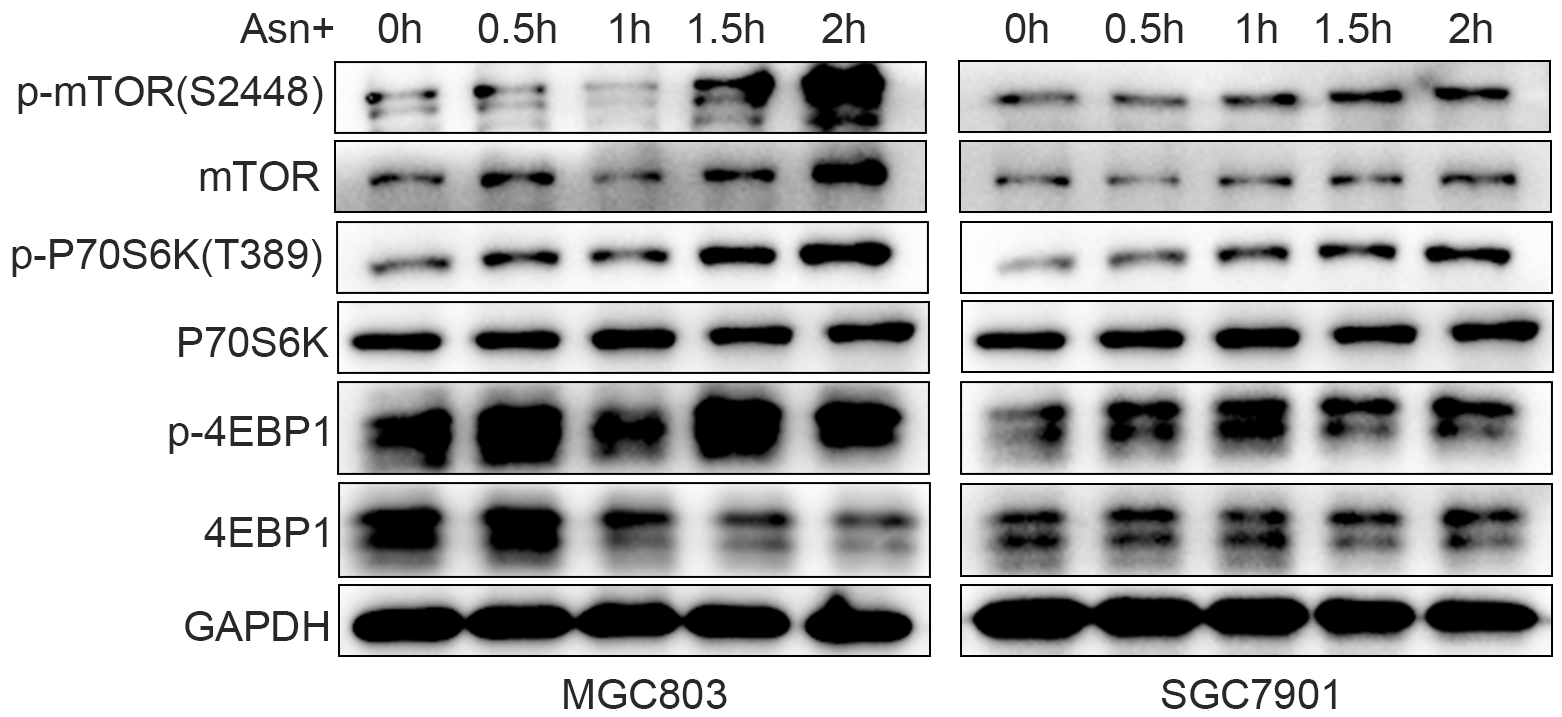

Supplement: Supplementary file 1 — Supplementary material 1: Figure S1 Asn independently activates the mTORC1 pathway. [file 10020_2024_1062_MOESM1_ESM.tif]

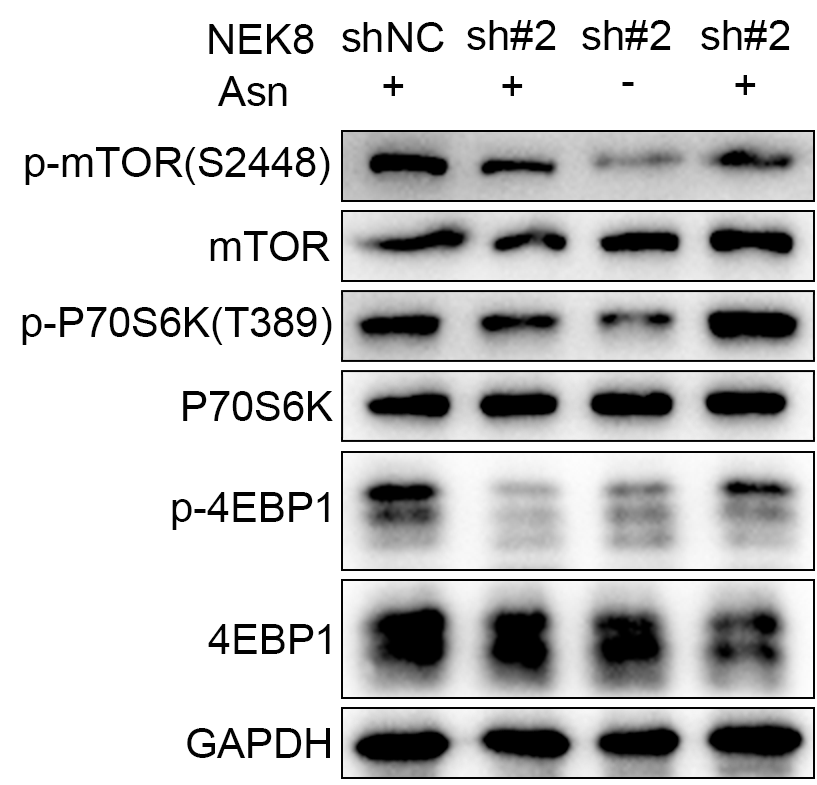

Supplement: Supplementary file 2 — Supplementary material 2: Figure S2 NEK8 regulates the mTORC1 pathway by modulating Asn metabolism. [file 10020_2024_1062_MOESM2_ESM.tif]
