# Supplementary material for: NEK8 promotes the progression of gastric cancer by reprogramming asparagine metabolism
Source: Mol Med. 2025 Jan 6;31:3. doi: 10.1186/s10020-024-01062-9 (PMC11702068; doi:10.1186/s10020-024-01062-9)
Supplement: Supplementary file 3 — Supplementary material 3. [file 10020_2024_1062_MOESM3_ESM.docx]

| Table S1 |  |  |  |  |  |  |
| --- | --- | --- | --- | --- | --- | --- |
| Variables | Univariate analysis | | | Multivariate analysis | | |
|  | HR | 95%CI | *P* | HR | 95%CI | P |
| Gender (male vs. female) | 1.256 | 0.655-2.410 | 0.496 | - | - | NA |
| Age (y) (<61 vs. ≥61) | 0.925 | 0.568-1.510 | 0.745 | - | - | NA |
| Tumor grades (I/II vs. III) | 0.569 | 0.343-0.943 | **0.029** | 0.740 | 0.436-1.256 | 0.264 |
| Tumor diameter (cm) (<6 vs. ≥6) | 1.677 | 1.005-2.776 | **0.048** | 1.144 | 0.684-1.911 | 0.609 |
| Differentiation | 0.271 | 0.124-0.596 | **0.001** | 0.337 | 0.151-0.753 | **0.008** |
| Location (upper vs middle vs.lower) | 0.865 | 0.636-1.140 | 0.271 | - | - | NA |
| Depth of invision (T1/TI vs. T3/T4) | 0.090 | 0.022-0.369 | **0.001** | 0.284 | 0.040-1.996 | 0.206 |
| Lymph node metastasis (yes vs. no) | 0.202 | 0.096.425 | **2.500E-5** | 0.786 | 0.060-10.329 | 0.855 |
| TNM stages (I/II vs. III/IV) | 6.800 | 3.433-13.830 | **1.210E-7** | 0.306 | 0.021-4.439 | 0.386 |
| NEK8 (positive vs. negative) | 2.476 | 1.472-4.164 | **0.001** | 0.440 | 0.259-0.748 | **0.002** |
|  |  |  |  |  |  |  |
